# Clinical and Radiological Analysis of Odontogenic Sinusitis: A Retrospective Study

**DOI:** 10.3390/jcm14082821

**Published:** 2025-04-19

**Authors:** Shin Hyuk Yoo, Hahn Jin Jung, Soo Kyoung Park, Ji-Hun Mo

**Affiliations:** 1 Department of Otorhinolaryngology, Dankook University College of Medicine, Cheonan 31151, Republic of Korea; shyoomd@gmail.com; 2 Department of Otorhinolaryngology-Head and Neck Surgery, Chungbuk National University Hospital, Chungbuk National University College of Medicine, Cheongju 28644, Republic of Korea; hahnjin2@naver.com; 3 Department of Otorhinolaryngology-Head and Neck Surgery, Chungnam National University Sejong Hospital, Chungnam National University College of Medicine, Daejeon 34134, Republic of Korea; pacsoo2@hanmail.net

**Keywords:** odontogenic sinusitis, endoscopic sinus surgery, dental implants, periodontal disease, computed tomography

## Abstract

**Background/Objectives**: Odontogenic sinusitis (ODS) is a distinct subtype of chronic rhinosinusitis that arises from dental pathology, with unique etiologies and treatment strategies. This study aimed to evaluate the clinical features, radiological findings, and surgical outcomes of ODS patients treated with endoscopic sinus surgery (ESS). **Methods**: This retrospective study included 139 patients diagnosed with ODS who underwent ESS. Demographic characteristics, clinical symptoms, radiological findings, and treatment outcomes were analyzed. Outcomes were assessed using pre- and postoperative LundKennedy (L-K) scores, with additional evaluation of the impact of concurrent dental treatment. **Results**: The most common etiology was periodontal disease (60.4%), followed by dental implants (20.1%) and dental extractions (19.4%). Radiological findings revealed sinus involvement beyond the maxillary sinus in 78.4% of patients, with common abnormalities including periapical abscesses (60.4%), oroantral fistulas (19.4%), and implant-related complications (20.1%). Patients who received concurrent dental treatment with ESS demonstrated significantly better outcomes, with success rates of 96.4% compared to 73.9% for those without dental treatment (*p =* 0.003). **Conclusions**: ODS is frequently underdiagnosed due to overlapping symptoms with chronic rhinosinusitis of other origins. Radiological imaging is crucial for identifying dental pathologies contributing to ODS. Combining ESS with dental treatment significantly improves outcomes and is recommended as the optimal management strategy for ODS.

## 1. Introduction

Odontogenic sinusitis (ODS) is a unique subset of chronic rhinosinusitis, often originating from dental pathologies. It accounts for approximately 10–12% of all sinusitis cases, yet it is frequently underdiagnosed due to nonspecific symptoms that overlap with other sinus diseases. ODS typically results from dental infections, implant complications, extractions, or other dental interventions that breach the Schneiderian membrane [1,2]. The growing number of dental procedures, including implants, has been associated with an increased recognition and diagnosis of odontogenic sinusitis (ODS) in recent years [3].

The microbiology and pathophysiology of ODS are distinct from other forms of rhinosinusitis. Dental pathogens, often anaerobic bacteria, play a critical role in its etiology [4,5]. Furthermore, the anatomical proximity of the maxillary teeth to the sinus floor makes the sinus susceptible to odontogenic infections [6]. Radiological imaging, particularly CT scans, is invaluable in diagnosing ODS, as it helps identify dental origins and extent of sinus involvement [7].

Despite its clinical significance, ODS is often misdiagnosed, leading to suboptimal management. Previous studies have highlighted the need for a multidisciplinary approach involving both otolaryngologists and dental professionals [8]. However, limited research has comprehensively characterized the clinical features, radiological findings, and outcomes of ODS treatment.

In this study, the authors carried out a retrospective study of 136 patients who had various causes of ODS to determine the clinical features, etiologic factors, radiological findings, and treatment outcomes.

## 2. Materials and Methods

### 2.1. Subjects

This study received ethical approval from the institutional review board of Dankook University Hospital (IRB No. 2019-04-010-002). A total of 139 patients diagnosed with ODS who underwent endoscopic sinus surgery (ESS) between January 2010 and July 2019 by a single surgeon at a tertiary referral hospital were included. All included patients demonstrated radiologic evidence of sinusitis with dental pathology in conjunction with endoscopic signs such as purulent discharge, mucosal edema, or nasal polyps on the ipsilateral side. All patients were refractory to at least 3 months of appropriate medical therapy, including antibiotics and other conservative treatments, and were considered for surgical intervention. Patients with other paranasal sinus lesions (e.g., tumors, odontogenic cysts, or fungal sinusitis) were excluded. All cases were consecutively included from a retrospective database of patients who underwent ESS for ODS between 2010 and 2019, minimizing selection bias. Detailed demographic characteristics, including age, sex, follow-up duration, and involved sinuses, are summarized in Table 1.

### 2.2. Clinical Characteristics

The etiology of ODS, including dental implants, dental extractions, or periodontal disease, was thoroughly assessed. Chief complaints, dental care history, and surgical outcomes were reviewed. Preoperative and postoperative Lund–Kennedy (L-K) scores were evaluated, with postoperative assessments conducted at least one month following surgery to ensure stability of results.

### 2.3. Lund–Kennedy Scoring System

The L-K score was used as a standard measure to evaluate the degree of inflammation and anatomical abnormalities observed during nasal endoscopy [9,10]. Each side of the nasal cavity was assessed, and the scores for both sides were summed to provide a total score ranging from 0 to 12. Higher scores reflected more severe disease. Specific components evaluated included mucosal edema, the presence of nasal polyps, and purulent discharge within the sinonasal cavity. Additional findings, such as scarring or crusting, were recorded when present. Preoperative and postoperative scores were assigned by the operating surgeon based on standardized grading criteria [9,11].

### 2.4. Radiological Evaluation

All patients underwent preoperative ostiomeatal unit computed tomography (OMU CT) imaging to identify sinus involvement, determine the etiologies of ODS, and locate problematic teeth. Radiological findings, such as periapical abscesses, oroantral fistulas, and implant-related abnormalities, were systematically analyzed [12,13]. The Lund–Mackay (L-M) scoring system was also employed to evaluate radiological severity. This validated scoring method assigns points for each sinus group (maxillary, ethmoid, frontal, sphenoid) based on the extent of opacification [14]. Scores range from 0 (no opacification) to 24 (complete opacification of all sinuses). Each side of the sinonasal cavity is graded independently, and the total score is calculated by summing the scores for both sides. Higher scores indicate more extensive disease.

### 2.5. Evaluation of Treatment Outcomes

All enrolled patients were treated with ESS. The surgical extent was tailored according to the extent of sinus involvement, as identified by CT imaging. To assess the role of dental care, patients were categorized into two groups: those who received concurrent dental treatment (dental treatment [+]) and those who did not (dental treatment [−]). All patients were recommended to undergo dental evaluation and appropriate treatment based on their individual radiologic and endoscopic findings. As part of our institutional protocol, a referral to a dental specialist was made for every patient. However, whether dental procedures were actually performed was left to the patient’s personal decision following dental consultation. Therefore, for the purpose of this retrospective analysis, patients were classified into two groups: those who received concurrent dental treatment and those who did not, based on treatment records. Preoperative and postoperative L-K scores were compared between these groups. Additionally, the impact of dental treatment on treatment outcomes for each etiology (periodontal disease, implants, and extractions) was analyzed. Patients were classified as having a “complete remission” if their ODS symptoms resolved, along with a total L-K score of 0.

For comparative analysis of treatment outcomes, the 3-month time point was used, as mucosal healing is typically stabilized at this stage, allowing for a reliable and standardized evaluation across patients.

### 2.6. Informe Consent Statement

Informed consent for publication was obtained from all identifiable human participants.

### 2.7. Statistical Analysis

All statistical analyses were performed using SPSS version 21.0 (IBM, Armonk, NY, USA). The Student’s *t*-test was used to compare mean preoperative and postoperative L-K scores between dental treatment (+) and dental treatment (−) groups. Prognosis comparisons were conducted using Fisher’s exact test. To evaluate factors influencing complete remission in ODS, a logistic regression analysis was performed. Data are presented as means ± standard deviation, with a two-sided *p* value < 0.05 considered statistically significant.

## 3. Results

### 3.1. Demographic Data and Clinical Characteristics

Data from 139 patients diagnosed with ODS were analyzed (Table 1). The median age of the participants was 51.9 years, ranging from 16 to 86 years. The cohort consisted of 84 males (60.4%) and 55 females (39.6%). The median follow-up duration for the patients was 10 months, with a range of 0.5 to 74.6 months.

The primary etiology for ODS was periodontal disease, accounting for 60.4% (*n* = 84) of cases. Iatrogenic causes, including dental implants (20.1%, *n* = 28) and dental extractions (19.4%, *n* = 27), collectively represented 39.6% of cases. These findings highlight the significant contribution of both periodontal and procedural dental origins to ODS.

The most commonly reported clinical symptom was purulent rhinorrhea, present in 36.0% (*n* = 50) of patients. Nasal obstruction was noted in 25.2% (*n* = 35), followed by foul odor in 17.3% (*n* = 24), cheek pain in 13.0% (*n* = 18), and postnasal drip in 6.5% (*n* = 9). A small proportion of patients (2.2%, *n* = 3) presented with other symptoms, including headache or facial swelling.

Endoscopic evaluations revealed a mean preoperative L-K score of 2.1 ± 0.4, indicating moderate disease severity. Following ESS, the mean postoperative L-K score significantly improved to 0.7 ± 0.6 (*p* < 0.01), reflecting the substantial resolution of sinonasal inflammation and associated symptoms.

Among all patients, a total of 55 individuals (39.4%) achieved complete remission, defined as the resolution of clinical symptoms, and a postoperative L-K score of 0.

**Figure 1:**
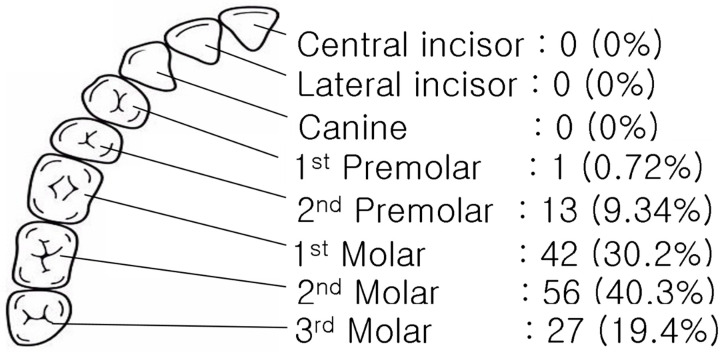
Evaluating clinical features and CT image findings, problematic teeth were identified.

Figure 1 illustrates the distribution of teeth implicated in ODS among the 139 patients. The second molars were the most frequently involved teeth, accounting for 40.3% (*n* = 56) of cases. This was followed by the first molars, implicated in 30.2% (*n* = 42) of cases, and the third molars, involved in 19.4% (*n* = 27). The second premolars were less frequently implicated, accounting for 9.3% (*n* = 13) of cases. Only one case (0.7%) involved the first premolar, and no cases involved the canines, lateral incisors, or central incisors. These findings highlight the anatomical proximity of the molar teeth to the maxillary sinus as a significant factor in the development of ODS.

### 3.2. Radiological Findings

CT imaging was performed for all 139 patients to evaluate the extent of sinus involvement and identify associated dental pathologies (Table 2). Sinus involvement was not limited to the maxillary sinus in most cases. Disease extended to the ethmoid sinuses in 37.4% (*n* = 52) of patients, the frontal sinuses in 38.9% (*n* = 54), and all sinuses in 2.2% (*n* = 3). Isolated maxillary sinus involvement was observed in only 21.6% (*n* = 30). Notably, ODS typically presents as a unilateral disease, which reflects its origin from localized dental pathology. This characteristic aids in distinguishing ODS from other forms of chronic rhinosinusitis and supports the importance of evaluating dental causes in cases of unilateral sinus opacification on imaging.

Additional findings on CT imaging highlighted key dental-related pathologies contributing to ODS. Periapical abscesses were present in 60.4% (*n* = 84) of cases, indicating advanced dental infections that had spread to the sinonasal region. Oroantral fistulas, a direct communication between the oral cavity and the maxillary sinus, were observed in 19.4% (*n* = 27) of patients. Improperly placed dental implants extending into the maxillary sinus were identified in 20.1% (*n* = 28) of cases, underscoring the role of iatrogenic factors in the development of ODS.

The mean preoperative L-M CT score was 3.7 ± 1.7, reflecting mild-to-moderate radiological severity.

### 3.3. Treatment Outcomes

Table 3 presents the correlation between preoperative and postoperative persistent symptoms with various clinical parameters, analyzed using multiple regression analysis. Patients who received concurrent dental treatment (dental Tx [+]) demonstrated significantly better postoperative outcomes compared to those who did not undergo dental treatment (dental Tx [−]). The mean postoperative Lund–Kennedy (L-K) score was significantly lower in the dental Tx (+) group (0.5 ± 0.6) compared to the dental Tx (−) group (0.9 ± 0.6, *p* < 0.001). This difference was primarily driven by reductions in mucosal edema (*p* < 0.001)

Logistic regression analysis was performed to identify factors influencing complete remission in patients with ODS. Table 4 presents the results of this analysis, highlighting significant and non-significant predictors of treatment outcomes. Among all variables analyzed, dental treatment emerged as the strongest predictor of complete remission. Patients who received concurrent dental treatment alongside ESS were significantly more likely to achieve complete remission compared to those who did not (odds ratio = 5.106, *p* = 0.001).

## 4. Discussion

ODS differs significantly from other chronic rhinosinusitis forms due to its unique etiology and management requirements [1,2]. This study demonstrates that ODS frequently involves multiple sinuses, with 78.4% of cases showing extension beyond the maxillary sinus. The second molars were most commonly implicated, highlighting their anatomical proximity to the sinus floor as a critical factor in the pathogenesis of ODS [2,7]. The findings emphasize the major role of dental procedures, such as implants and extractions, as significant contributors to ODS [6,15].

CT imaging emerged as a critical diagnostic tool, enabling the accurate identification of dental etiologies such as periapical abscesses, oroantral fistulas, and implant-related abnormalities [5]. These results align with existing recommendations for routine imaging in cases of unilateral sinus disease to avoid diagnostic errors and guide effective treatment. Radiological findings also facilitated the localization of problematic teeth, which was crucial for planning comprehensive management strategies [4,8].

Combining ESS with dental interventions resulted in significantly improved outcomes, demonstrating the necessity of addressing the underlying dental pathology to minimize recurrence and promote long-term recovery [1,4,8]. Prognostic benefits were consistent across various ODS etiologies when dental care was integrated, reinforcing the importance of a multidisciplinary treatment approach [15]. This finding reinforces the necessity of a multidisciplinary approach, integrating dental care with ESS to optimize ODS treatment outcomes.

This study contributes to the growing body of evidence advocating for collaboration between otolaryngologists and dental professionals in managing ODS. Enhanced interdisciplinary communication can improve diagnostic accuracy, particularly in cases of atypical or complex presentations, and reduce delays in initiating appropriate therapy. Additionally, the role of patient education should not be overlooked. Informing patients of the potential risks associated with untreated dental conditions may help prevent the progression to ODS.

Nevertheless, this study has limitations. As a single-center retrospective analysis, the findings may not be universally applicable. Additionally, variability in follow-up durations poses challenges in assessing long-term outcomes. Future research should employ standardized protocols and include multicenter data to validate these results [2,5]. Further research should also explore the cost-effectiveness of routine dental evaluations for patients presenting with unilateral sinus disease, as well as the utility of advanced imaging modalities, such as cone-beam CT, in diagnosing early-stage ODS. Investigations into optimal surgical techniques and perioperative care protocols may also yield valuable insights into improving patient outcomes.

The increasing prevalence of ODS, driven by the rise in dental procedures, underscores the need for targeted strategies to address this growing health concern. By integrating dental care with rhinologic interventions and leveraging advancements in diagnostic and therapeutic technologies, clinicians can enhance patient outcomes and mitigate the burden of ODS on healthcare systems [6,16]. These findings reinforce the necessity of a comprehensive, multidisciplinary approach to effectively manage this condition.

## 5. Conclusions

This study highlights the critical importance of multidisciplinary care in managing ODS. Radiological assessments and dental interventions combined with ESS significantly enhance clinical outcomes. Addressing both sinus and dental pathologies ensures better prognoses and reduces recurrence rates. Standardized diagnostic criteria and exploration of long-term impacts are essential for future progress.

## Figures and Tables

**Table 1:** Demographical and clinical characteristics of the study patients.

Variable	Total Patients (*n* = 139)
Median age (range), years	51.9 (16–86)
Sex, *n* (%)	
Male	84 (60.4)
Female	55 (39.6)
Follow-up duration (range), months	10.0 (3.5–74.6)
Etiology, *n* (%)	
Periodontal disease	84 (60.4)
Iatrogenic causes	55 (39.6)
Dental implant	28 (20.1)
Dental extraction	27 (19.4)
Clinical symptoms, *n* (%)	
Purulent rhinorrhea	50 (36.0)
Nasal obstruction	35 (25.2)
Foul odor	24 (17.3)
Cheek pain	18 (13.0)
Postnasal drip	9 (6.5)
Others	3 (2.2)
L-K endoscopic score (mean ± SD)	
Preoperative	2.1 ± 0.4
Postoperative	0.7 ± 0.6
Complete remission of ODS, *n* (%)	55 (39.6)

Abbreviations: L-K, Lund–Kennedy; ODS, odontogenic sinusitis.

**Table 2:** CT image findings of study patients.

Variable	Total Patients (*n* = 139)
Involved sinus, *n* (%)	
Maxillary sinus	30 (21.6%)
Maxillary, ethmoid sinus	52 (37.4%)
Maxillary, ethmoid, frontal sinus	54 (38.9%)
All sinuses	3 (2.2%)
L-K endoscopic score	
Preoperative	2.1 ± 0.4
Postoperative	0.7 ± 0.6
Preoperative L-M CT score (mean ± SD)	3.7 ± 1.7
Additional CT findings, *n* (%)	
Periapical abscess	84 (60.4)
Dental implants	28 (20.1%)
Oroantral fistula	27 (19.4%)

Abbreviations: CT, computed tomography; L-K, Lund–Kennedy; L-M, Lund–Mackay.

**Table 3:** Comparison of preoperative Lund–Mackay and pre- and postoperative Lund–Kennedy scores based on dental treatment.

Variable (Mean ± SD)	Dental Treatment (+) (*n* = 76)	Dental Treatment (−) (*n* = 63)	*p* Value
Lund–Mackay score (preoperative)	3.8 ± 1.7	3.7 ± 1.8	
Lund–Kennedy score			
Preoperative (total)	2.1 ± 0.4	2.0 ± 0.4	
Edema score	1.2 ± 0.4	1.3 ± 0.5	
Polyp score	0.1 ± 0.2	0.1 ± 0.3	
Discharge score	0.9 ± 0.4	0.7 ± 0.4	
Postoperative (total)	0.5 ± 0.6	0.9 ± 0.6	<0.001
Edema score	0.4 ± 0.5	0.9 ± 0.4	<0.001
Polyp score	−	−	
Discharge score	0.1 ± 0.2	0.1 ± 0.4	

**Table 4:** Multivariate logistic regression analysis of prognostic factors for complete remission in odontogenic rhinosinusitis.

Variables	B	S.E.	Wald	*p* Value	Exp (B)
Sex	0.380	0.500	0.576	0.448	1.462
Age	0.029	0.017	2.976	0.085	1.030
Site	−0.497	0.863	0.331	0.565	0.608
Nasal polyp	0.487	1.132	0.185	0.667	1.627
Oroantral fistula	0.553	2.054	0.073	0.788	1.739
Dental implant	0.541	2.054	0.069	0.792	1.717
Preapical abscess	0.241	2.054	0.014	0.907	1.273
Dental treatment	1.630	0.491	11.017	0.001	5.106

## Data Availability

The original contributions presented in this study are included in the article. Further inquiries can be directed to the corresponding author.
